# The Influence of PVDF Membrane Ageing on the Efficiency of Bacterial Rejection During the Ultrafiltration Treatment of Carwash Wastewater

**DOI:** 10.3390/ma19020324

**Published:** 2026-01-13

**Authors:** Piotr Woźniak, Marek Gryta

**Affiliations:** Faculty of Chemical Technology and Engineering, West Pomeranian University of Technology in Szczecin, Al. Piastów 17, 70-310 Szczecin, Poland; piotr.wozniak@zut.edu.pl

**Keywords:** ultrafiltration, carwash wastewater, membrane ageing, bacteria, water reuse, fouling

## Abstract

This study investigated the influence of two years of ultrafiltration (UF) on the separation properties of tubular polyvinylidene fluoride membranes used for treating carwash wastewater, particularly with regard to bacterial rejection. Fouling was mitigated by washing the membranes with alkaline cleaning agents (pH > 11.5). Repeated applications of these agents enlarged the membrane pores to approximately 300 nm. This affected bacterial retention, and for feed containing bacteria (determined as colony-forming units, CFU) at a concentration of 3.11 × 10^6^ CFU/mL, over 13,000 CFU/mL were detected in the permeate. Interestingly, fouling improved retention, reducing bacterial counts present in the permeate from 13,689 to 2889 CFU/mL. Fouling also enhanced the retention of surfactants (80%), chemical oxide domain (60%), and turbidity (below 0.5 NTU), yielding results comparable to new membranes. Daily 60-min membrane washing with Wheel Cleaner solution (pH = 11.5) improved the membranes performance; however, it did not remove deposits from large pores, allowing good rejection performance and a permeate flux of 65 LMH to be maintained. It was found that bacteria also developed on the permeate side. Disinfection of the module housing with a NaOH/NaOCl solution reduced the number of bacteria in the permeate from 5356 to 66 CFU/mL. Microbiological tests revealed that some of these bacteria were antibiotic-resistant.

## 1. Introduction

The car wash industry produces large volumes of wastewater, which poses significant the environmental and operational challenges [1]. The worst-case scenario is the discharge of this wastewater directly into surface water bodies, which leads to severe environmental degradation. This typically occurs in countries where only a small percentage of industrial and municipal wastewater is treated [2]. An alternative approach is to implement wastewater treatment that enables the recovery of water for various applications [3,4,5]. The treatment of carwash wastewater (CWW) is primarily implemented in automatic car washes, enabling the recycling of over 60% of the used water [5,6,7].

The composition of CWW is complex and it usually contains surfactants, suspended solids, oil, grease, salts, heavy metals, polycyclic aromatic hydrocarbons and microorganisms [8]. Due to this diverse composition, CWW requires multiple treatment processes [3]. Advanced methods such as flocculation-flotation, coagulation, electrocoagulation and membrane separation are used to enhance the efficiency of conventional treatment methods such as sedimentation and sand filtration [3,5,9]. These multi-stage treatment systems are commonly implemented in large-scale car washes; however, their high cost limits their use in smaller facilities.

Low-cost treatment and economic feasibility have been achieved through the use of nature-based solutions for treating and reusing carwash wastewater [4,6]. In such systems, CWW is directed through constructed wetlands, which require a substantial land area. Although these solutions are effective, their implementation in urban environments is challenging due to space limitations.

Small, self-service car wash stations equipped with pressure guns are widely used in European cities, with several thousand operating in Poland alone. Drivers select the process that best suits their needs, which typically includes pre-washing, foam washing, rinsing, waxing and polishing with permeate from the reverse osmosis process. These stations are supplied with tap water and the resulting wastewater is collected in a settling tank before being discharged into the municipal sewage system via an oil separator. Previous studies have shown that ultrafiltration (UF) can recover up to 70% of the washing water from such CWW [10]. The UF process effectively removes oils, greases and suspended solids, consistently producing a permeate with turbidity below 0.3 NTU regardless of the initial wastewater composition [10,11]. Furthermore, it has been confirmed that using reclaimed water for car washing does not affect the quality of automotive coatings [12].

Carwash wastewater also contains microorganisms that can be retained by UF membranes [13]. However, the effective separation of these microorganisms remains challenging, and bacteria such as *Cryptosporidium* and *Giardia* are frequently detected in recycled water [14,15,16]. Therefore, it is essential to monitor the microbiological quality of water in car washes that utilise recycling systems [17]. Additionally, the high-pressure flow of the solution during the car washing promotes aerosol formation. These aerosols may contain pathogenic microorganisms that have been washed off the vehicle surface and thus pose a potential health risk to users [18,19,20]. Nevertheless, pressure guns are typically used for only a few minutes at self-service wash stations, and this short contact time significantly reduces the risk of infection [21]. However, bacteria present in CWW can accumulate on membrane surfaces, leading to biofouling that impairs the performance of UF systems [22,23,24].

It is well known that the formation of deposits on membrane surfaces (fouling) can significantly hinder the industrial implementation of membrane processes [22,25,26,27]. The intensity of fouling depends on the composition of the feed and the type of membrane material used. Tubular polyvinylidene fluoride (PVDF) membranes have been successfully used to separate CWW [10]. PVDF membranes are also widely used in other industrial applications [28].

It has been demonstrated that fouling caused by CWW can be mitigated by periodically cleaning the membranes with alkaline solutions (pH > 11.5) commonly used for car washing [22,29,30]. Although alkaline cleaning is often used for PVDF membranes, the long-term application may increase their pore size [31,32]. However, during CWW ultrafiltration, these larger pores become blocked by fouling relatively quickly, which limits deterioration in the separation efficiency of suspended solids and surfactants [29].

During the long-term operation of the module, the exposure of the PVDF membranes to the feed water and cleaning agents, as well as the accumulation of irreversible foulants, leads to membrane ageing [22,28,29,33]. Obviously, this process affects membrane performance, with a deterioration in *Escherichia coli* retention observed after five years of UF module operation [30]. However, the most of UF studies were conducted in a laboratory setting, which may have influenced the ultrafiltration results [28]. Therefore, this work reports results obtained from a pilot plant equipped with an industrial module containing tubular PVDF membranes. It should be expected that small washers will operate membrane modules in a manner different from that recommended by membrane manufacturers. In the present study, the UF module was operated for two years in CWW treatment experiments, incorporating shutdown periods and membrane washing with agents typically used for car cleaning. The study aimed to evaluate the effect of this operational period on membrane performance, particularly in terms of bacterial retention. Due to the potential health risk posed by bacteria passing into the permeate, the study also investigated the antibiotic resistance of bacteria present in the UF system used for CWW filtration.

## 2. Materials and Methods

### 2.1. UF Pilot Plant

**Figure 1:**
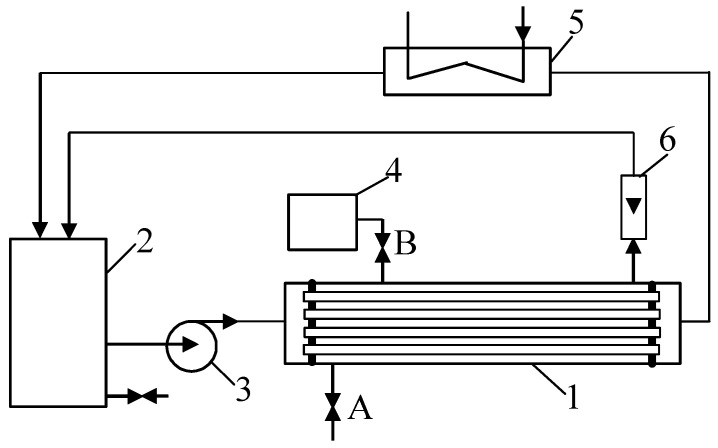
UF pilot plant. 1—tubular module, 2—feed tank (150 L), 3—centrifugal pump, 4—cleaning agent tank, 5—water cooling, 6—rotameter, A, B—valve.

To investigate the impact of membrane degradation on UF process performance, the tubular PVDF membranes that had been used for two years to separate CWW in a pilot plant were employed in this study. A schematic diagram of the experimental setup is shown in Figure 1. The installation was equipped with a B1 module containing 18 tubular FP100 membranes (PCI Membranes, Kostrzyn Wielkopolski, Poland). The internal diameter of the tubes was 12.5 mm, and the total membrane surface area was 0.9 m^2^. The specifications of the FP100 membranes are presented in Table 1.

The pilot plant construction details and the course of the two-year CWWs separation study are presented in a previous publication [29]. During the studies, the membranes were repeatedly chemically cleaned using alkaline solutions (pH > 11.5). At the beginning of the UF testing degraded membranes, one FP100 tubular membrane was replaced with a new one to allow for sampling and analysis of membrane ageing.

A 100 L of feed solution was prepared for the UF tests. The feed flowed through the interior of the tubular membranes at a velocity of 1.9 m/s, and the UF process was carried out at a transmembrane pressure (TMP) equal to 0.1 MPa. Ultrafiltration of CWW was carried out for several hours per day, and was followed by membrane washing.

In addition to the regular cleaning of the feed side of the pilot plant, the permeate side of the UF module was also cleaned. For chemical cleaning of the module housing, the permeate was first drained from the system (Figure 1, valve A open, rotameter (6) disconnected). Next, the space between the membranes was filled by gravity with a solution of an alkaline professional cleaning agent (TESOL ME—RADEX, Kołbaskowo, Poland) supplied from tank 4 (valves: A—closed, B—open). After 10 min, the TESOL ME solution was removed, and the housing was rinsed twice with nanofiltration permeate.

In addition to bacterial rejection studies, separation tests for surfactants and chemical oxygen demand (COD) were also performed. Rejection efficiency (R) was determined as follows:

(1) $$R\;[\%]=\frac{C_{F}-C_{P}}{C_{F}}100$$

where C_F_ [mg/L] and C_P_ [mg/L] are the measured concentrations of the feed and permeate, respectively.

### 2.2. Working Solutions

The permeate from the nanofiltration (NF) process, which used tap water as its feed, was applied to rinse the UF pilot plant and prepare the feed solutions (Table 2). Microbiological tests confirmed the absence of bacteria in the NF permeate. The NF permeate was also used to determine the water permeate flux value, which is expressed in litres per square metre per hour (L/m^2^h) and denoted as LMH.

As in the previous UF studies, synthetic CWW prepared from concentrates of cleaning agents used in car washes was applied for the UF experiments [29]. The prepared CWW contained 0.5 vol.% Euro Turbo Foam Color Blue foaming agent and 0.2 vol.% Euro Blue Wax solution (both from EuroEcol, Łódź, Poland). This mixture included surfactants, diethylene glycol butyl ether, 5,5′-indigodisulfonic acid sodium salt, benzenesulfonic acid, 4-C10-13-sec-alkane sodium salts, and soluble polymers. For this type of wastewater, the values of the analysed parameters were [mg/L]: chemical oxygen demand (COD) 3110 ± 250, anionic surfactants 410 ± 30, and nonionic surfactants 35 ± 12. The turbidity of the prepared CWW varied during UF and ranged between 220 and 240 NTU.

Car wash owners prefer to use the same cleaning agents for membrane washing as those used for washing vehicles. For this reason, a 0.5 vol.% alkaline solution (pH = 11.5) of Wheel Cleaner (EuroEcol, Łódź, Poland) was used for membrane washing. The solution contained NaOH, ethylenediaminetetraacetic acid tetrasodium salt, sulfonic acids, C14–16-alkane hydroxy and C14–16-alkene sodium salts, diethylene glycol butyl ether, and 1-Propanaminium,3-amino-N-(carboxymethyl)-N,N-dimethyl-,N-(C12–18 even-numbered acyl) derivatives [34].

Before weekends or longer shutdowns, the UF installation was rinsed twice with NF permeate and then flushed with 100 L of a 0.25% sodium metabisulphite (Na_2_S_2_O_5_) solution (ChemLand, Stargard, Poland). A 0.5 vol.% solution of cleaning agent TESOL ME (RADEX, Kołbaskowo, Poland) was used to disinfect the module housing. This solution contains NaOH and NaOCl. The concentrate of this agent contained 12–18% active chlorine.

### 2.3. Analytical

The calorimetric Hach cuvette tests (Hach Lange, Düsseldorf, Germany) were used to determine the concentration of surfactants (LCK 333—nonionic, LCK 432—anionic), and COD (LCK 1014).

The composition of the solutions was determined using a 940 Professional IC Vario ion chromatograph (Metrohm AG, Herisau, Switzerland).

The turbidity of tested solutions was measured with a portable turbidity meter model 2100 AN IS (Hach Company, Loveland, CO, USA).

The changes in the membrane morphology were observed using a scanning electron microscope—SEM (SU8020, Hitachi, Tokyo, Japan).

### 2.4. Microbiological Studies

Over the course of two years of CWW separation research [29], the UF installation became contaminated with bacteria originating from the car wash environment. Despite the membranes being repeatedly cleaned with chemicals, some bacteria survived and proliferated in the tested solutions. For the microbiological analysis, bacteria were isolated from the feed solution of the UF installation operating in a closed-loop system for a minimum of 2 h.

Bacterial growth was carried out on Petri dishes using culture media supplied by BioMaxima (Lublin, Poland). For total viable counts, a non-selective medium (plate count agar) was used in accordance with standard procedures. After 24 h of incubation, the number of colony-forming units (CFU) was counted. Each culture was prepared in triplicate and the results reported as mean CFU/mL values.

Species-level identification was performed by mass spectrometry using the Bruker MALDI Biotyper^®^ (MBT; Bruker Daltonik GmbH, Bremen, Germany). Individual bacterial colonies were transferred to Columbia LAB-AGAR^TM^ Base, a non-selective medium (BioMaxima, Lublin, Poland), to establish pure cultures. These bacteria isolates were spotted onto a metal target plate for MALDI-TOF/MS examination and allowed to dry following matrix application. The mass spectrum were matched against the IVD MBT reference library to determine bacterial species.

The Bruker MALDI Biotyper enabled the identification of bacterial species present in wastewater, for which data for taxonomic analysis are available from the National Center for Biotechnology Information (NCBI) database (https://www.ncbi.nlm.nih.gov/, accessed on 10 September 2025). Sequences of the 16S rRNA gene, retrieved from NCBI/EZtaxon/Ribosomal Database Project (accessed on 10 September 2025), were employed to generate a phylogenetic tree of detected bacteria. Sequence similarities were performed using the nucleotide BLAST (blastn) (http://www.ncbi.nlm.nih.gov/BLAST/, accessed on 10 September 2025). All sequence were alignments conducted with Clustal W, and phylogenetic relationships were inferred by constructing a maximum likelihood (ML) tree with MEGA software version 12.

### 2.5. Antibiotic Resistance Testing

The antibiotic resistance test of detected bacteria was performed using the EUCAST standardised disk diffusion method (https://www.eucast.org, accessed on 7 October 2025). Susceptibility discs Oxoid^TM^ (Thermo Fisher Scientific, Basingstoke, UK) were used for the tests. The kinds of applied antibiotics were presented in Table 3.

**Figure A1:**
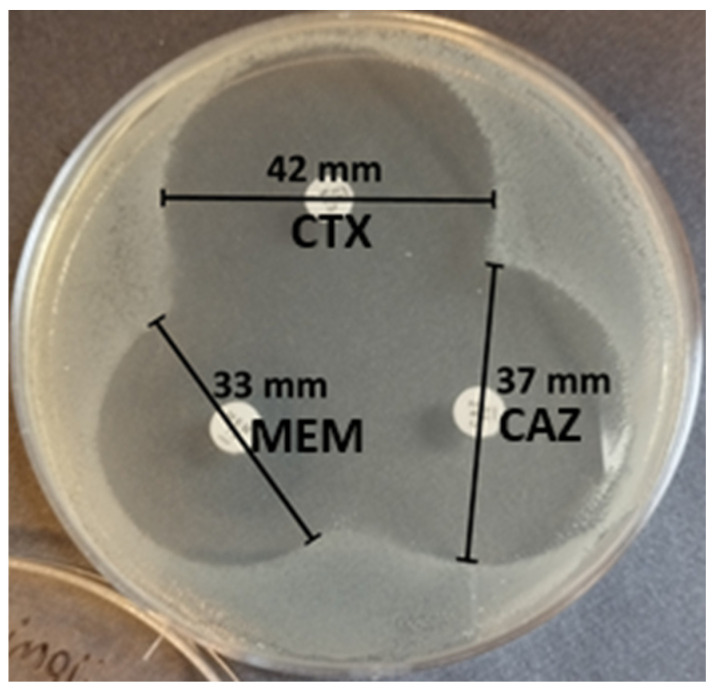
Inhibition zones after 24 h of incubation of *Phytobacter ursingii* for antibiotics CTX, MEM, and CAZ (Table 1).

Pure bacterial colonies were isolated from the bacteria present in the analysed CWW. These colonies obtained from an overnight culture were collected with a sterile swab and were suspended in a test tube containing physiological saline and mixed until a uniform turbidity was achieved. The bacterial concentration was estimated based on absorbance measurement at a wavelength of 625 nm. The absorbance of the bacterial suspension should be within the range of 0.08–0.13, which corresponds to 1–2 × 10^8^ CFU/mL. The bacterial solution was spread evenly over the entire plate. The drug impregnated disks were firmly pressed onto the surface of an inoculated agar plate. The bacterial growth was carried out at 36 °C for 24 h. The diameters of the inhibition zones were measured with a ruler to the nearest 1 mm (Figure A1). The zone diameters were then converted into susceptibility categories according to the current breakpoint tables available at https://www.eucast.org/clinical_breakpoints, (accessed on 7 October 2025). The assessment of the impact of sample zone diameter values is presented in Table A1.

## 3. Results

### 3.1. Membrane Structure

Following two years of CWW separation research, as presented in [29], the UF pilot installation was preserved using a 0.25% sodium metabisulphite solution. After a two-week shutdown, the system was flushed twice with 100 L of NF permeate and a sample of the FP100 membrane was collected for analysis.

**Figure 2:**
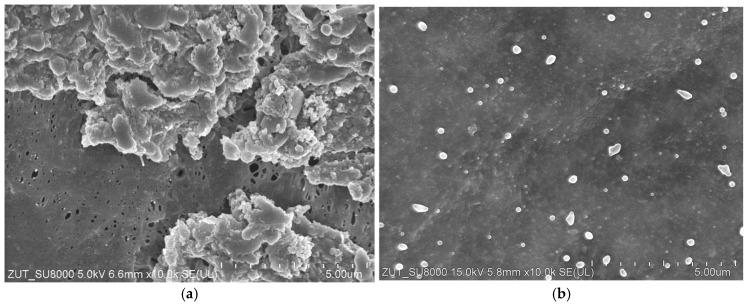
SEM images of internal surface FP100 membrane: (**a**) with deposits after two years of CWW separation studies with cyclic alkaline washing, (**b**) virgin membrane.

SEM observation revealed that despite repeated chemical cleaning throughout the two-year research period, deposits remained on the membrane surface (Figure 2a). In areas free from deposits, pores ranging from 100 to 300 nm in dimension were observed. These pores were significantly larger and more numerous than those found on virgin FP100 membranes (Figure 2b). The increase in pore size confirmed earlier findings that alkaline cleaning induces structural changes in PVDF membranes [29,30].

Although NaOH solutions are commonly used to mitigate membrane fouling, these results highlight the importance of proceeding with caution in industrial applications as PVDF membranes demonstrate limited resistance to prolonged chemical exposure [31,32].

**Figure 3:**
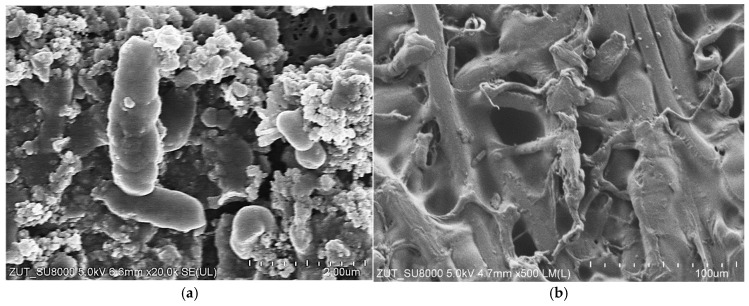
SEM image of tubular FP100 membrane: (**a**) deposit on the internal surface containing numerous bacterial cells, (**b**) external surface (supporting layer).

Bacterial cells were identified within the deposits observed on the membrane surfaces (Figure 3a). The majority of these microorganisms were rod-shaped bacteria. Their presence indicates that the UF installation was colonised by bacterial strains that were resistant to the alkaline cleaning agents, which were applied repeatedly during the two-year CWW filtration process [29]. In contrast to the internal PVDF layer, only minor deposits were visible on the outer surface of the tubular membranes (Figure 3b).

Although NaOH solutions are known to destroy bacterial cells, some strains can adapt to high-pH environments and survive in them [35]. More than 30 bacterial species were previously identified in wastewater collected from the tested car wash facility. Prior studies have shown that certain strains can persist within the UF system despite the use of aggressive chemical agents and elevated temperatures (333 K) during membrane cleaning [24]. This resilience is attributed to the formation of protective biofilms and to hard-to-clean areas in industrial installations, such as pump piston seals and valves, where cleaning agents have limited access. Consequently, bacterial survival resulted in severe biofouling, even when the UF system was supplied exclusively with distilled water [24].

In industrial installations, bacterial growth is usually controlled using strong disinfectants. However, the proposed process of CWW treatment cannot use such aggressive agents in UF systems designed for small car washes. Car wash owners generally oppose the use of chemicals that could potentially damage vehicle paintwork. Moreover, CWW contains a wide variety of bacterial species, and their continuous presence must be considered in relation to the long-term operation of the UF module. Persistent microbial activity can lead to biofouling, negatively affecting membrane performance and system efficiency.

### 3.2. CWW Ultrafiltration

**Figure 4:**
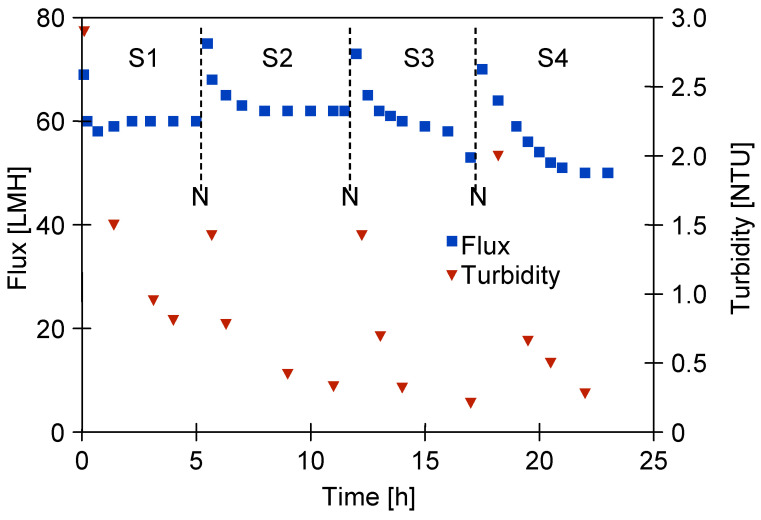
Changes in permeate flux and turbidity during CWW separation. N—the feed remained in the module during the overnight break; subsequently, the membranes were washed with a 0.5% Wheel Cleaner solution (pH = 11.5). S1–S4—UF series.

After two years of UF module operation, the water permeate flux was measured at 233 LMH. Following the addition of CWW components to the feed water, this value dropped significantly to 60 LMH (Figure 4, series S1). The UF process was conducted over several hours, after which the module was kept filled with CWW for the overnight break.

**Figure A2:**
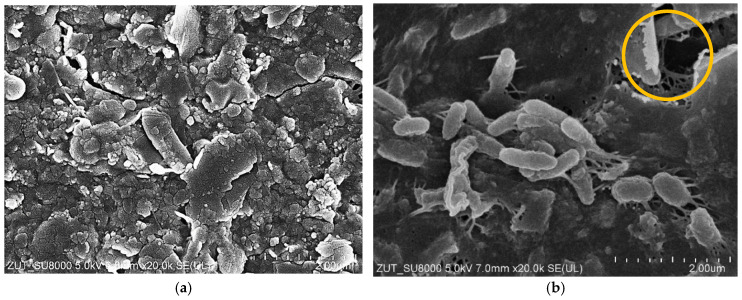
SEM images of FP100 membranes covered by deposits: (**a**) after 10 h filtration of CWW, (**b**) after 30 min washing fouled membrane with 0.5 vol.% Wheel Cleaner solution (pH = 11.5). Circle—large pores cleaned as a result of membrane washing.

SEM analysis confirmed that the observed decline in permeate flux was caused by deposits forming on the membrane surface (Figure A2a). To reduce fouling prior to the next experimental series, the CWW was removed from the system after the night break and the module was rinsed for 30 min with a Wheel Cleaner solution (pH = 11.5). The UF process was then resumed by reintroducing the tested CWW into the pilot plant.

It is necessary to mention that following membrane cleaning, the amount of surface deposits decreased (Figure A2b) and the permeate flux increased to 75 LMH. However, after one hour of UF operation, the flux decreased again to 60 LMH (Figure 4).

During UF testing, the feed turbidity ranged from 220 to 240 NTU. Following the chemical cleaning of the membranes, the permeate exhibited a turbidity of 2–3 NTU, which decreased quickly and stabilised below 0.5 NTU (Figure 4). This suggests that the larger pores, cleaned previously with the Wheel Cleaner solution (Figure A2b), were quickly blocked by newly formed deposits, limiting the permeation of feed components through the membrane. These findings suggest that membrane fouling can improve separation performance. Consequently, minor defects in the membrane structure (Figure 2a) will not impair the retention of suspended solids, as was also observed in previous studies [29].

**Figure 5:**
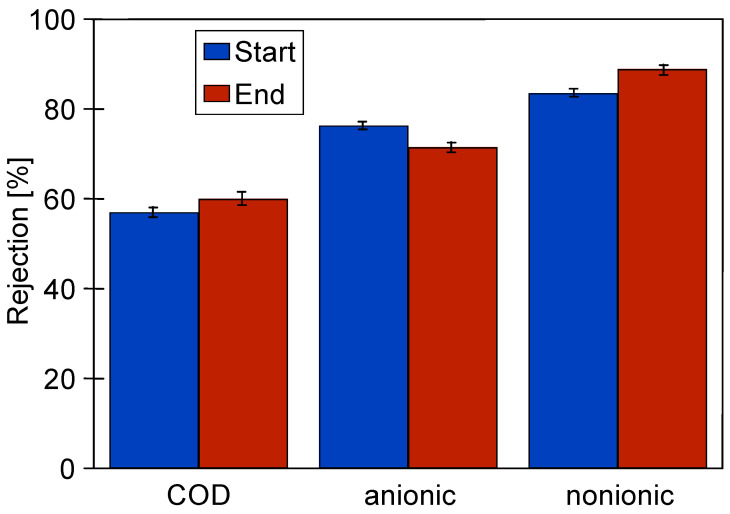
Changes in the rejection of CWW components during UF studies at the beginning (Start) and after three weeks of pilot plant operation (End).

The formation of a fouling layer also had a beneficial effect on the retention of solutes present in CWW, such as surfactants. After 10 h of UF operation, the rejection rate for surfactants reached 80%, while COD removal was approximately 60% (Figure 5, Start). Similar values were obtained after an additional three weeks of UF testing, as described in subsequent sections of this paper (Figure 5, End).

The positive impact of the fouling layer is further supported by the observation that the retention rates for COD and surfactants were similar to those achieved with the use of new FP100 membranes [10,29]. This suggests that the fouling layer can compensate for minor structural defects in the membrane, which enhances separation performance. However, an increase in pore size, as observed after two years of membrane operation (Figure 2a), may reduce the membrane’s ability to reject bacteria. To investigate whether membrane fouling could have a compensatory effect, the influence of fouling on bacterial retention was examined.

During the first hour of UF testing following system maintenance with a sodium metabisulphite solution, the bacterial concentration in the feed was 347 CFU/mL, whereas the permeate contained only 26 CFU/mL. The sodium metabisulphite solution used for maintenance releases free chlorine, which helps to suppress bacterial growth during system downtime.

However, after 21 h of CWW separation testing (Figure 4, series S4), the bacterial count in the feed, which was used continuously over four days, had increased to 3.11 × 10^6^ CFU/mL, with 235 CFU/mL detected in the permeate. This substantial increase indicates that the composition of the tested CWW strongly promotes bacterial proliferation. The bacterial load in the feed was comparable to that found in real car wash wastewater [36]. Such intense bacterial growth may explain the more pronounced decline in permeate flux observed on the fourth day of UF testing (Figure 4, series S4).

**Figure 6:**
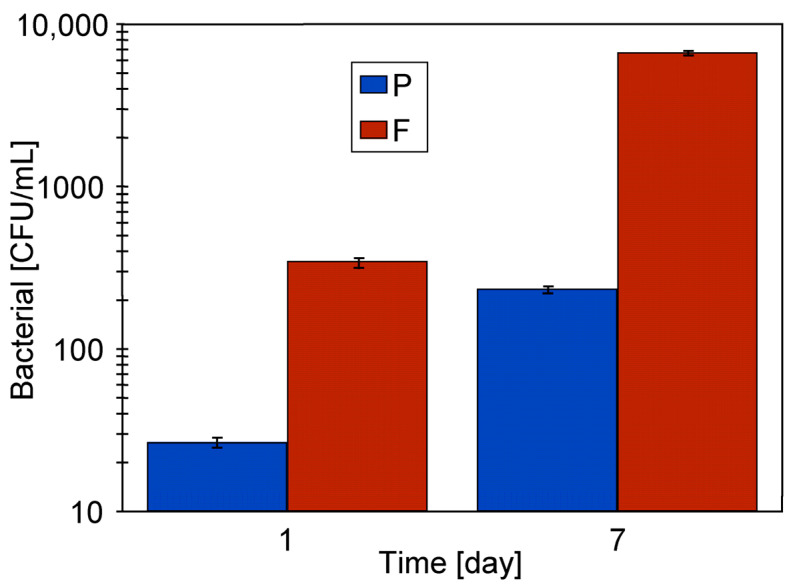
Change in bacterial count in the feed (rinsing water) and permeate at the beginning and end of the UF study (Figure 4). P—permeate, F—feed.

After completing the S4 series, the installation was flushed with water and subsequently cleaned with a 0.5% Wheel Cleaner solution for 30 min. The UF module was then emptied. Following the weekend break, the system was flushed with 100 L of water, and UF operation was resumed using NF permeate as the feed. After 40 min of water recirculation, the permeate flux reached 195 LMH, but later decreased to 145 LMH. This decline suggests the formation of a fouling layer, likely caused by bacteria present in the feed (6711 CFU/mL) (Figure 6, 7 days). The presence of bacteria in the recirculated rinsing water was probably due to biofilms being dislodged from the internal surfaces of the UF installation. As demonstrated in previous studies, such biofilms are difficult to remove and can promote bacterial growth even when the UF system is supplied with distilled water [24].

### 3.3. Effect of Washing Time with Wheel Cleaner Solution

**Figure 7:**
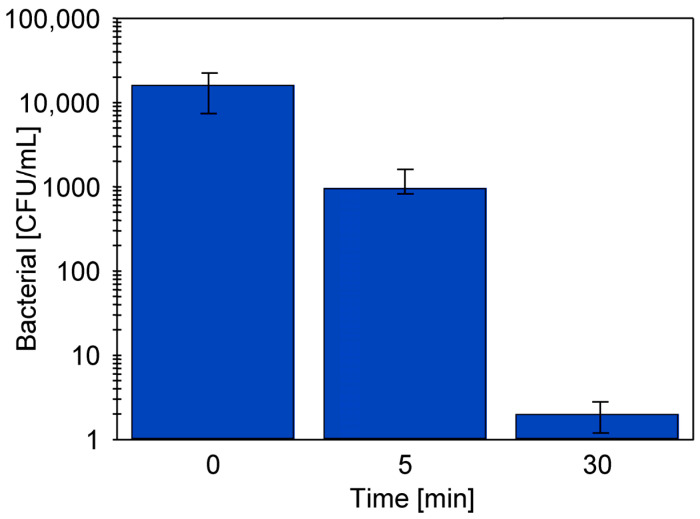
Change in the number of bacteria in a 0.5% Wheel Cleaner solution (pH = 11.5). Initially, 10 mL of CWW containing bacteria (1.6 × 10^6^ CFU/mL) was added to 1 L of the Wheel Cleaner solution).

In a previous study, it was shown that daily 30-min rinsing of membranes with an alkaline solution helped to reduce fouling during CWW separation [22]. The 0.5% Wheel Cleaner solution (pH = 11.5) also effectively eliminated most of the live bacteria present in the tested UF system. After 30 min of contact with this solution, the number of bacteria decreased from 1.61 × 10^4^ to 2 CFU/mL (Figure 7).

**Figure 8:**
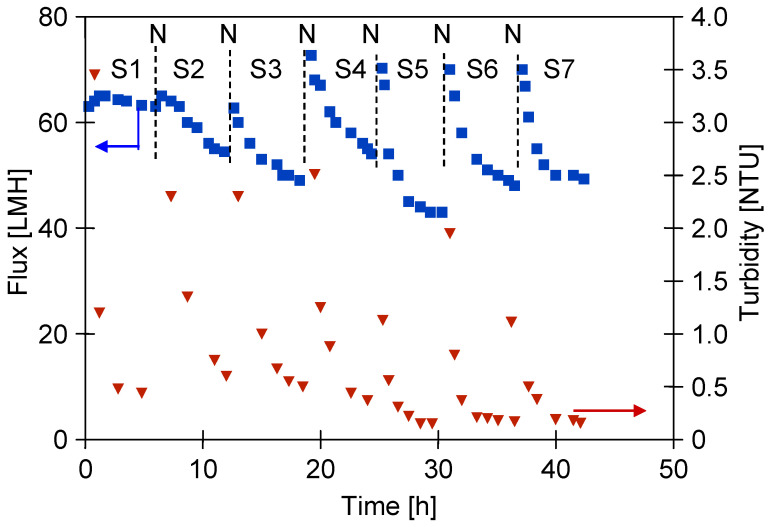
Changes in permeate flux and turbidity during CWW separation. N—overnight pauses during which the module was filled with a 0.5% Wheel Cleaner solution (pH = 11.3–11.5). S1–S7—UF series.

However, the results presented in Figure 4 indicate that this may be insufficient to prevent a decline in permeate flux if intensive bacterial growth occurs in the UF system. Although the Wheel Cleaner solution significantly reduced the number of bacteria, numerous microorganisms were still observed on the membrane surface after the cleaning agent had been recirculated through the UF system for 30 min (Figure A2b). To improve the efficiency of membrane cleaning, the pump was turned off after the 30-min washing period and the Wheel Cleaner solution was left in the UF system overnight. Following this pause, the module was rinsed with water, after which the UF process for the tested CWW was resumed. As shown in Figure 8, extending the contact time with the alkaline solution did not eliminate fouling. However, after this operation, there was stabilisation of changes in the permeate flux, which decreased repeatedly from 70 to 45–50 LMH over the three-day testing period.

**Figure 9:**
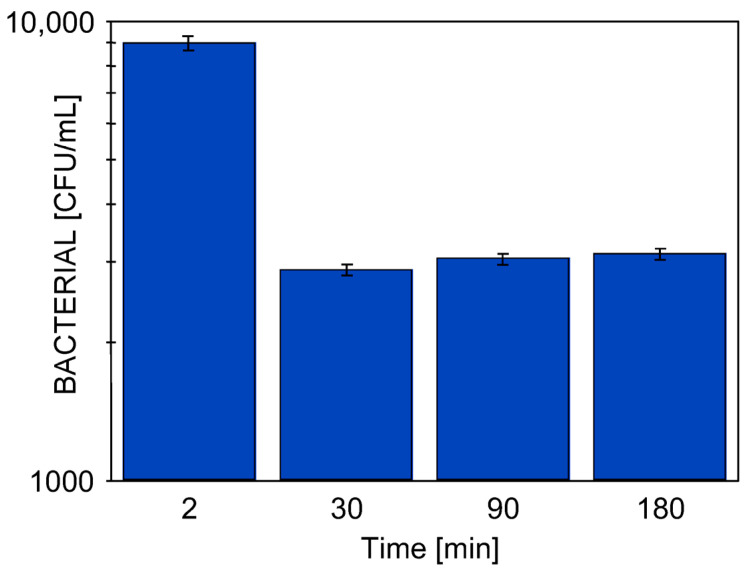
Effect of CWW separation time resumed after membrane washing (0.5% Wheel Cleaner) on the bacterial content in the resulting permeate. Bacterial concentration in the feed: 1.03 × 10^7^ CFU/mL.

Performed alkaline cleaning of the membrane removes contaminants from the interior of the larger pores (Figure 2a and Figure A2a). As a result, during the initial minutes of the UF process, the turbidity of the permeate was 2.5–3 NTU. However, due to fouling, the pores became blocked and the turbidity quickly dropped to below 0.2 NTU (Figure 8). Previous studies have shown that the formation of a fouling layer on the membrane surface can significantly affect the outcomes of CWW separation [29]. On the sixth day of UF testing (Figure 8, S6), the impact of fouling on bacterial retention was investigated. For this purpose, the UF installation was thoroughly cleaned and then fed with CWW containing 1.03 × 10^7^ CFU/mL of bacteria. During the first five minutes of UF, a high number of bacteria (13,689 CFU/mL) were detected in the permeate, which decreased to 2889 CFU/mL after 30 min of UF process (Figure 9). Over the following hours, this value increased slightly to 3133 CFU/mL. These results confirmed previous findings on *E. coli* separation using FP100 membranes, where nearly 100% bacterial retention was achieved after 30 min of UF due to fouling [30]. This effect was also observed in the case of membranes that had been in UF operation for five years; however, due to extensive damage to the active layer, complete bacterial retention was not achieved.

**Figure 10:**
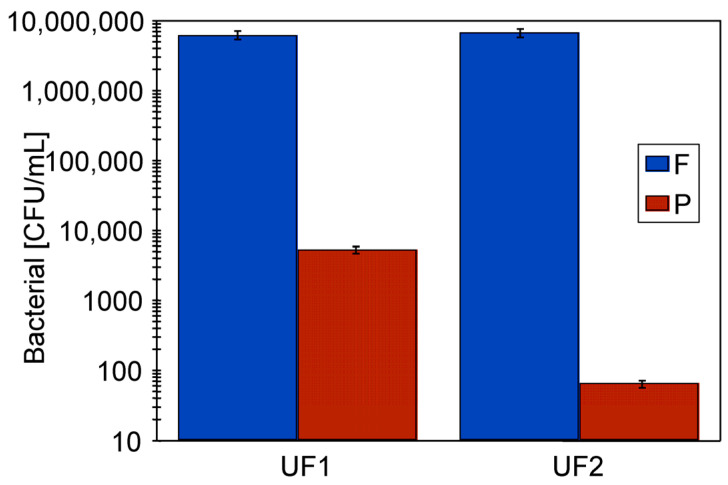
Effect of tubular module housing sterilisation on the bacterial content in the resulting permeate. UF1—before, and UF2—after 10-min module washing with 0.5% TESOL ME solution. F—feed, P—permeate.

The passage of bacteria into the permeate can contaminate the module housing, potentially promoting bacterial growth on the clean side of the UF system. Therefore, the high number of bacteria detected in the permeate (Figure 9, after 30 min) may result not only from their passage through the membranes, but also from the release of bacteria growing within the module housing. To demonstrate this, after two hours of CWW separation containing 6.29 × 10^6^ CFU/mL of bacteria (Figure 8, series S7), a permeate sample was collected, which contained 5356 CFU/mL. The pump in the UF system was then turned off (TMP = 0), and module B1 was sterilised on the permeate side. For this purpose, the module housing was first rinsed with water and then filled with a 0.5% TESOL ME solution, which released free chlorine. After ten minutes, the solution was removed, and the housing was rinsed twice with water. The CWW separation process was then resumed (TMP = 0.1 MPa), and a permeate sample was taken after 30 min of UF. Analysis of the bacterial content showed a reduction from 5356 to 66 CFU/mL (Figure 10). This result confirms that the previously obtained permeate samples may have been secondarily contaminated by bacteria growing in the module housing.

**Figure 11:**
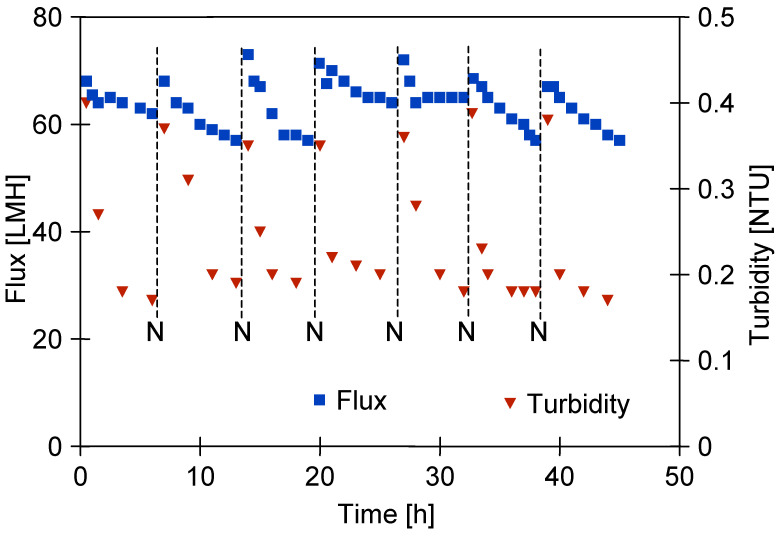
Changes in permeate flux and turbidity during CWW separation with cyclic 60-min membrane washing after night break (N) using Wheel Cleaner solution.

The results obtained (Figure 9) suggest that increased fouling results in greater bacterial retention. Therefore, cleaning the membranes too thoroughly may not be beneficial. The main objective in this case is to limit fouling in order to maintain an acceptable permeate flow rate. For this reason, in the next stage of UF testing, the membranes were no longer soaked overnight in the Wheel Cleaner solution; instead, the membranes were washed for 60 min. Consequently, the permeate flux stabilised at 65 LMH and the turbidity of the permeate remained below 0.2 NTU (Figure 11). Immediately after washing the membranes, the turbidity was 0.4 NTU, which is significantly lower than that observed after overnight soaking (Figure 8, after N). These results suggest that the washing method (60 min) did not completely remove deposits from the interior of the larger pores, which may help restrict bacterial penetration through the membranes.

**Figure 12:**
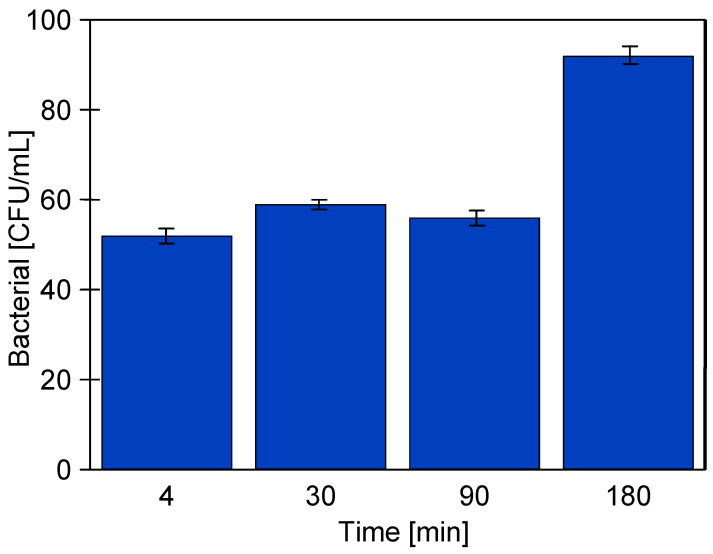
Effect of tubular module housing sterilisation on bacterial content in the permeate during CWW separation with a 60-min membrane washing with Wheel Cleaner solution.

To demonstrate the effect that a longer membrane washing period has on bacterial separation, module B1 (the feed side) was washed with Wheel Cleaner for 60 min. Additional, as in previous tests, the module housing was then sterilised with a TESOL ME solution. After thoroughly rinsing the UF system with water, the separation of the CWW solution (7.19 × 10^6^ CFU/mL) was resumed. The initial bacterial content in the permeate (52 CFU/mL) was similar to that obtained for fouled membranes (Figure 9, UF2). This noteworthy result indicates that, even after 60 min of washing, deposits blocking the larger pores remained on the membrane surface. The bacterial content in the permeate increased to 92 CFU/mL after 180 min (Figure 12). This finding indicates that a small number of bacteria passing through the membrane can lead to growth on the permeate side.

### 3.4. Results of Microbiological Studies

**Figure 13:**
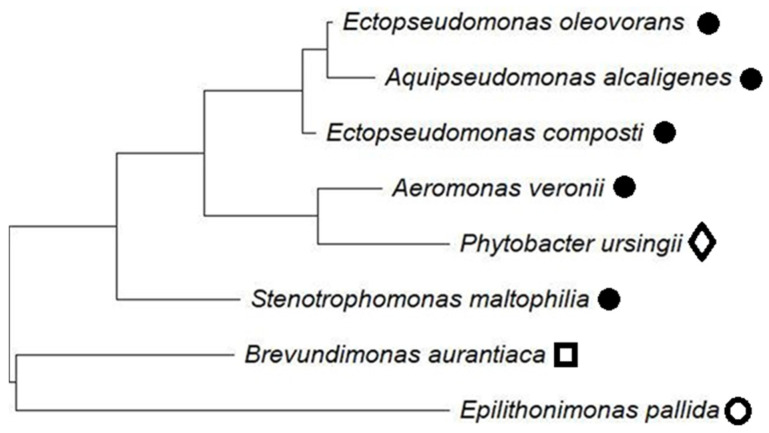
The phylogenetic tree of the bacteria identified in the feed. Groups: ●—γ-proteobacteria, ◊—enterobacteria, ○—CFB group bacteria, □—α-proteobacteria.

The present research confirmed that UF permeate obtained from carwash wastewater may contain bacteria due to membrane ageing. Analysis of bacterial species present within the UF pilot installation revealed the development of eight species during the CWW separation process (Figure 13). Although microbiological indicators such as *Escherichia coli* and *Legionella* spp. are commonly monitored in reused water [4], they were not detected in the tested samples.

Some of the bacteria identified, including *Brevundimonas aurantiaca*, *Stenotrophomonas maltophilia*, *Aeromonas veronii*, and *Aquipseudomonas alcaligenes*, are recognised as causative agents of challenging human infections [37,38,39,40]. *Stenotrophomonas maltophilia* is a globally emerging, multidrug-resistant opportunistic pathogen [41]. In this study, it exhibited resistance to ceftazidime (CAZ), while its growth was inhibited by tobramycin (TOB) and meropenem (MEM) (Table 4). Its ability to colonise device surfaces and form biofilms enhances its resistance to both phagocytes and antibiotics [38]. Colonisation and infection with *Stenotrophomonas maltophilia* are often managed using broad-spectrum antimicrobials, including carbapenems, third-generation cephalosporins (e.g., ceftazidime and cefepime), and quinolones [38].

In the study conducted, *Brevundimonas aurantiaca* exhibited the highest level of resistance to the antibiotics applied. Consistent with findings reported in previous studies, this species was resistant to ciprofloxacin (CIP) but not to levofloxacin (LEV) [37]. The previously reported lack of resistance of *B. aurantiaca* to carbapenems was only partially confirmed in this study, resistance was absent for meropenem (MEM), but present for imipenem (IPM) (Table 4).

Studies on *Aeromonas* bacteria inhabiting aquatic environments have shown that these strains can metabolise carbohydrates, amino acids, carboxylic acids, fatty acids, and saturated hydrocarbons. The bacteria were found to adhere to PVC surfaces and form biofilms in which they multiplied intensively [42]. Such bacteria may therefore thrive in carwash wastewater (CWW) and form biofilms on the surfaces of treatment installations.

## 4. Conclusions

Alkaline cleaning agents used in car washing can also effectively reduce fouling on PVDF ultrafiltration membranes during carwash wastewater treatment. Daily cleaning (60 min) with Wheel Cleaner (pH ≈ 11.5) maintained high flux (≈65 LMH) and good separation performance over two years.

However, prolonged exposure to NaOH solutions caused membrane aging and pore enlargement up to approximately 300 nm. Despite the formation of such large pores, industrial-scale performance was strongly influenced by fouling, which enhanced the retention of surfactants, COD, and turbidity, achieving results comparable to those of new membranes.

Complete bacterial retention was not achieved due to membrane aging. Although fouling improved separation efficiency, small quantities of bacteria still passed into the permeate, reaching concentrations of approximately 50 CFU/mL. Consequently, bacterial growth occurred on the permeate side, significantly increasing the bacterial load in the recovered washing water. Periodic sterilization of the permeate side using a sodium hydroxide and sodium hypochlorite solution was found to mitigate this phenomenon.

Despite regular chemical cleaning of the UF installation, eight bacterial species were identified within the system. Some of these, such as *Brevundimonas aurantiaca*, exhibited resistance to various antibiotics. Therefore, sterilization of the reused water before its application in car washing is essential.

## Figures and Tables

**Table 1:** The specifications of the FP100 membranes.

Parameter	Unit	Data
Membrane-forming polymer	-	PVDF
Classification	-	UF
MWCO	kDa	100
Maximum process pressure	MPa	1.0
Maximum operating temperature	K	353
Water contact angle	deg	<80
pH range	-	1.5–12

**Table 2:** Concentration of major ions in the tap water and NF permeate [mg/L].

Ion	Na^+^	K^+^	Ca^2+^	Mg^2+^	NO_3_^−^	Cl^−^	SO_4_^2−^
Tap water	25.41	6.12	60.11	17.615	0.89	49.20	91.10
NF permeate	1.81	0.35	0.82	0.28	0.05	11.04	0.94

**Table 3:** Types of antibiotics used in antibiotic resistance testing.

Antibiotic	Symbol	Disk Content (µg)	Antibiotic Class
Cefepime	FEP	30	Cephalosporins
Ceftazidime	CAZ	30	Cephalosporins
Cefotaxime	CTX	30	Cephalosporins
Ciprofloxacin	CIP	5	Fluoroquinolones
Levofloxacin	LEV	5	Fluoroquinolones
Tobramycin	TOB	10	Aminoglycosides
Imipenem	IPM	10	Carbapenems
Meropenem	MEM	10	Carbapenems
Trimethoprim-sulfamethoxazole	SXT	25	Miscellaneous agents

**Table 4:** Antibiotic susceptibility of bacteria identified in UF treated carwash wastewater: susceptible (S) or resistant (R). “na”—indicates that the parameter was not tested because the antibiotic is not recommended for treating infections caused by the given type of bacteria.

Bacteria	FEP	CAZ	CTX	CIP	LEV	TOB	IPM	MEM	SXT
*Ectopseudomonas oleovorans*	S	S	S	S	S	S	S	na	na
*Aquipseudomonas alcaligenes*	S	S	S	S	S	S	S	na	na
*Ectopseudomonas composti*	S	S	S	S	S	S	S	na	na
*Aeromonas veronii*	na	S	na	S	na	S	na	S	na
*Stenotrophomonas maltophilia*	na	R	na	na	na	S	na	S	na
*Phytobacter ursingii*	S	S	na	na	S	S	S	na	S
*Brevundimonas aurantiaca*	R	R	S	R	S	R	R	S	S
*Epilithonimonas pallida*	R	S	na	na	R	S	S	na	S

## Data Availability

The original contributions presented in the study are included in the article; further inquiries can be directed to the corresponding author.

## Abbreviations

The following abbreviations are used in this manuscript:

FEP	Cefepime
CAZ	Ceftazidime
CTX	Cefotaxime
CIP	Ciprofloxacin
LEV	Levofloxacin
TOB	Tobramycin
IPM	Imipenem
MEM	Meropenem
SXT	Trimethoprim-sulfamethoxazole

## Appendix A

Antibiotic resistance testing was performed using the EUCAST standardized disk diffusion method, which involves determining the zone diameter around a disk impregnated with an antibiotic (Figure A1). The measured values indicate whether a given bacterial strain is resistant (R) or susceptible (S) to the tested antibiotics. Example results are presented in Table A1.

**Table A1:** The influence of zone diameter values [mm] on the classification as resistant (R) or susceptible (S) to the tested antibiotics in selected bacterial species. “na”—antibiotic not recommended for the treatment of infections caused by this bacterial species. Data collected from tables available at https://www.eucast.org/clinical_breakpoints (accessed on 7 October 2025).

Antibiotic	*Pseudomonas* spp.		*Enterobacteriales*		*Aeromonas* spp.	
	S≥	R<	S≥	R<	S≥	R<
FEP	50	21	27	24	27	24
CAZ	50	17	27	24	24	21
CTX	na	na	20	20	na	na
CIP	50	26	25	22	na	na
LEV	50	18	23	19	27	24
TOB	18	18	16	16	na	na
IPM	50	20	22	19	na	na
MEM	24	18	22	16	na	na
SXT	na	na	14	11	19	16
